# Palliative care in the education of physiotherapists in Germany - an anonymized cross-sectional survey of trainees and students

**DOI:** 10.1186/s12909-025-08205-4

**Published:** 2025-11-06

**Authors:** Anna Elisabeth Pape, Alisa Schmitt, Tom Militzer, Sandra Apelt, Paula Vradelis, Christian Volberg

**Affiliations:** 1 Department of Therapy Somatic, Klinikum Bremen-Ost, Bremen, Germany; 2 Department of Anaesthesiology & Intensive Care Medicine, Faculty of Medicine, Philipps-University of Marburg, Marburg, Germany; 3 Department of Physiotherapy, University Hospital of Marburg, Marburg, Germany

**Keywords:** palliative care, physiotherapy, education, training, end-of-life care, educational content

## Abstract

**Background:**

Physiotherapy plays a pivotal role in the comprehensive care of patients, including those in palliative circumstances. Despite the recognized importance of palliative care competencies, there is evidence that this content may not be adequately integrated into physiotherapy education curricula.

**Methods:**

To address this gap in the literature, a quantitative study was conducted using a structured online questionnaire to collect data from physiotherapy trainees and students in Germany. The questionnaire contained 30 questions, focusing on demographic data, educational details, prior knowledge and experience, educational content, practical experience, and personal attitudes and preparation. The collected data underwent thorough analysis, incorporating both descriptive statistics and more in-depth investigation into specific hypotheses concerning the integration of palliative care into physiotherapy education.

**Results:**

A total of 305 physiotherapy trainees and students participated in the survey. Most of the participants identified as female (70.5%), and 75.2% of them were in training. Furthermore, it was found that 71.8% were under the age of 25. While 76.6% of respondents were able to define palliative care, only 41.9% had received teaching on this topic as part of their training. A significant proportion of the participants (89.8%) expressed a strong interest in additional educational material in this domain. Furthermore, 51.6% of respondents reported having gained practical experience with palliative clients. However, a notable proportion of respondents (52.5%) reported experiencing stress in their work with palliative clients.

**Conclusion:**

The findings underscore a conspicuous incongruity between the necessity for and the accessibility of palliative care education within the physiotherapy curriculum. While a rudimentary understanding of palliative care is evident, it is apparent that many students have not received adequate training in this domain. The pronounced interest among students in additional teaching content and practical experience underscores the necessity for enhanced integration of palliative care education into physiotherapy curricula.

**Supplementary Information:**

The online version contains supplementary material available at 10.1186/s12909-025-08205-4.

## Background

Palliative medicine has gained increasing importance in recent decades, particularly in the context of caring for patients afflicted with oncological and chronic, incurable diseases. The fundamental objective of palliative care is to enhance patients’ quality of life by alleviating pain and other distressing symptoms, while concurrently addressing their psychosocial and spiritual needs. Physiotherapy, in particular, assumes a pivotal role in this context, offering benefits that extend beyond mere pain management and mobility enhancement. Its integration into healthcare services not only alleviates physical discomfort but also fosters independence and improves overall well-being among patients [1, 2].

On an international level, there is an increasing focus on the integration of palliative care in the training of healthcare professionals. Interdisciplinary curricula, exemplified by those implemented in Canada, underscore the pivotal role of physiotherapists within multidisciplinary palliative care teams [3, 4]. The integration of education and training into a systematic framework has been demonstrated to enhance students’ knowledge and practical skills, thereby improving the quality of patient care [5].

Notwithstanding the central importance of palliative care in healthcare, there is evidence that palliative care content is not sufficiently integrated into physiotherapy education in Germany [6–8]. This deficiency may result in future physiotherapists lacking the optimal preparation for the unique challenges and requirements associated with providing care to palliative patients. Inadequate training in this area can adversely impact both the quality of therapy and the well-being of the therapists themselves, as working with seriously ill and dying patients can be emotionally stressful [9, 10].

Furthermore, extant studies have demonstrated that an important proportion of physiotherapy students and trainees harbor sentiments of insecurity regarding their competencies and proficiencies in palliative care. This underscores the pressing need for a comprehensive review and potential expansion of the educational curriculum to ensure that students are well-equipped with the requisite competencies to competently engage in palliative care [6, 8]. In Germany, only individuals who have successfully completed their training are given the title of ‘physiotherapist’. Training takes place at vocational schools for trainees and at universities for students.

The rationale underlying the present survey was to cultivate a more profound comprehension of how physiotherapy trainees and students are being prepared for their future role in palliative care. The survey, which was designed to assess current training content, knowledge and experience, and attitudes towards palliative care, aims to identify gaps in training and make recommendations for enhanced curricular integration. This initiative is poised to ensure that future physiotherapists possess not only the willingness but also the capacity to make a substantial contribution to palliative care, thereby leading to a sustainable enhancement in the quality of life of those affected.

## Objective

The present study aims to develop a comprehensive understanding of how physiotherapy trainees and students are prepared for their future role in palliative care. This includes several specific objectives:

1. Survey of the state of education: Determine the integration of palliative care in physiotherapy education programs to demonstrate how this content is currently integrated into curricula.
2. Assessment of knowledge: Analysis of trainees’ knowledge related to palliative care to identify potential knowledge gaps.
3. Analysis of practical experience: Examining students’ experiences with palliative clients to understand the extent to which they have already acquired practical knowledge in this area.
4. Identification of training needs: Identify student’s needs and preferences regarding teaching content in palliative care to find out which topics are considered particularly relevant.
5. Promotion of curricular development: Development of recommendations for improved curricular integration of palliative care into physiotherapy training to ensure that students are optimally prepared for their future tasks.

## Methods

### Study design

This study used a quantitative cross-sectional survey among physiotherapy students and trainees in Germany. A quantitative approach was chosen to systematically capture the prevalence of palliative care content in curricula, the level of knowledge and practical experience, and attitudes among a large group of participants. The STROBE guideline was used as a reporting system for this survey.

### Recruitment

Participants were recruited from physiotherapy training centers and universities in Germany. The objective of the study was to establish a comprehensive database, which was constructed based on the voluntary participation of the respondents.

### Data collection instrument

The data were collected using a structured online questionnaire, which was developed by the study team in collaboration with specialists from the field of physiotherapy and palliative medicine following intensive literature research on the subject area. The questionnaire is divided into different sections:

1. Demographic data:
  Gender.
  Age.
  Training or studies to become a physiotherapist.
2. Training details:
  Year of training or semester of studies.
3. Previous knowledge and experience:
  Knowledge about palliative medicine/palliative care.
4. Training content:
  Lessons on the topic of palliative care.
5. Practical experiences:
  Experience and fields of work with palliative patients.
6. Personal attitudes and preparation:
  Personal attitude, preparation and resilience when working with palliative patients.

Following the pilot phase, the final questionnaire comprised 30 items and took about 15 min to complete. Participants indicated agreement or disagreement using Likert scales, single- and multiple-choice questions, with follow-up items triggered by filter questions. To maximize completeness, all items were mandatory; respondents could not proceed without answering. For multiple-choice questions, selecting a single option was sufficient to continue.

Furthermore, the PCEP-GR questionnaire (The Program in Palliative Care Education and Practice Questionnaire, German revised version) was utilized to ascertain the sentiments of trainees when providing care to the terminally ill [11]. The raw version of the questionnaire is available online as a supplement.

### Data acquisition

The data presented herein were obtained through the implementation of an online survey, administered via the survey platform surveymonkey.com and distributed to physiotherapy trainees and students in Germany. A request was disseminated by email on a nationwide scale to the respective academic institutions (*n* = 14) at which physiotherapy is taught. The relevant training centres (*n* = 255) were also asked to forward the survey invitation to trainees and students. The information on the individual training centres is based on data obtained from the German Physiotherapy Association. To encourage participation, reminders were sent via email on February 26, 2024, and April 24, 2024, to all physiotherapy schools and universities. Furthermore, a public call for participation was issued on 22.04.2024 (Instagram) and 05.04.2024 (Newsletter) via “Physio Deutschland” (German Physiotherapy Association). The data acquisition was conducted anonymously to protect the privacy and confidentiality of the participants. The survey was closed on June 1, 2024, after a total duration of 14 weeks. The predefined selection responses were analysed purely descriptively, while the responses from the free text fields were first categorised according to common themes and then analysed. The analysis was conducted utilising the Microsoft Excel version 16.100.1 software.

### Ethics and data protection

The study was approved by the responsible ethics committee of the medical faculty of the Philipps-University of Marburg on November 21, 2023 (file number: 23–287 ANZ) and registered in the German Clinical Trials Register (DRKS00033464). All participants were informed about the purpose of the study in a cover letter, and their participation was voluntary. The study adhered to the prevailing data protection regulations in Germany, and the collected data were anonymized to ensure that no conclusions could be drawn about the participants.

## Results

### Recording the level of education

A total of 305 participants completed the survey. Participants were included if they were currently enrolled in a physiotherapy program and provided informed consent; no other exclusion criteria were applied.

The mean age of respondents was 25.6 years (SD 6.5 years). Most participants were female (70.5%). The majority were engaged in training (75.2%), while 24.8% were pursuing academic studies. The predominant proportion of trainees surveyed was in their second year of education (42.7%) and the largest proportion of students was in their sixth semester (27.0%) (see Table 1).

**Table 1:** Demographic data of the participants

	*n*	%
Gender		
female	215	70.5
male	86	28.2
divers	4	1.3
Age (Years)		
< 25	219	71.8
25–35	60	19.7
36–45	16	5.2
46–55	5	1.6
> 55	5	0.7
Qualifications		
Training	224	75.2
1. Training year	50	22.7
2. Training year	95	42.7
3. Training year	76	24.5
Study	74	24.8
1. Semester	7	9.5
2. Semester	10	13.5
3. Semester	5	6.8
4. Semester	14	18.9
5. Semester	7	9.5
6. Semester	20	27.0
7. Semester	2	2.7
8. Semester	7	9.5
9. Semester	1	1.3
10. Semester	1	1.3
Experience with palliative clients		
Yes	147	51.6
No	138	48.4

### Assessment of knowledge

Most respondents (76.6%) reported that they were able to define palliative care, indicating a basic understanding of the field. However, a fifth of respondents (21.0%) expressed uncertainty regarding the definition of palliative care. A minimal percentage (2.4%) admitted a lack of familiarity with the term’s definition. Furthermore, 89.7% of respondents reported familiarity with the term “hospice.” However, only 41.9% reported having received palliative care instruction as part of their formal training.

###  Analysis of practical experience

Regarding practical experience, 51.6% of respondents had previously worked with palliative patients, with the most common locations being hospital wards outside a palliative care unit (70.7%) and palliative care units (42.0%). Hospices accounted for 3.3% of the responses, while retirement and nursing homes constituted 10.0%. Within physiotherapy practice, 7.3% of respondents reported having worked with palliative patients, while 4.6% indicated that they had done so in the form of home visits (outside of specialized outpatient palliative care teams). A significant proportion of the participants, 52.6%, reported experiencing stress in their work with palliative patients, while 43.1% reported finding it beneficial.

###  Identification of training needs

The satisfaction levels concerning the teaching content on palliative care were found to be mixed in this study. A total of 41.9% of the respondents indicated that they had previously received instruction on palliative care in the classroom setting. The largest proportion of respondents (39.8%) received instruction on the topic during their second year of training, followed by 17.1% in the first year and 10.6% in the third year. The largest proportion of students (14.6%) received instruction on palliative care in the third semester, and the smallest proportion (1.6%) in the first semester. The remaining semesters exhibited equal distribution, with 3.3% and 4.1% of students in each semester. Notably, a majority of 89.8% expressed a keen interest in pursuing further knowledge in the domain of physiotherapy within the context of palliative care.

### Personal attitude and preparation

The survey results indicate that 19.1% of the participants expressed a high level of interest in the domain of palliative care, while 44.3% indicated a certain degree of affinity with this subject area. Moreover, 81.2% of the respondents asserted the imperative of incorporating palliative care as an integral component of professional training.

**Fig. 1:**
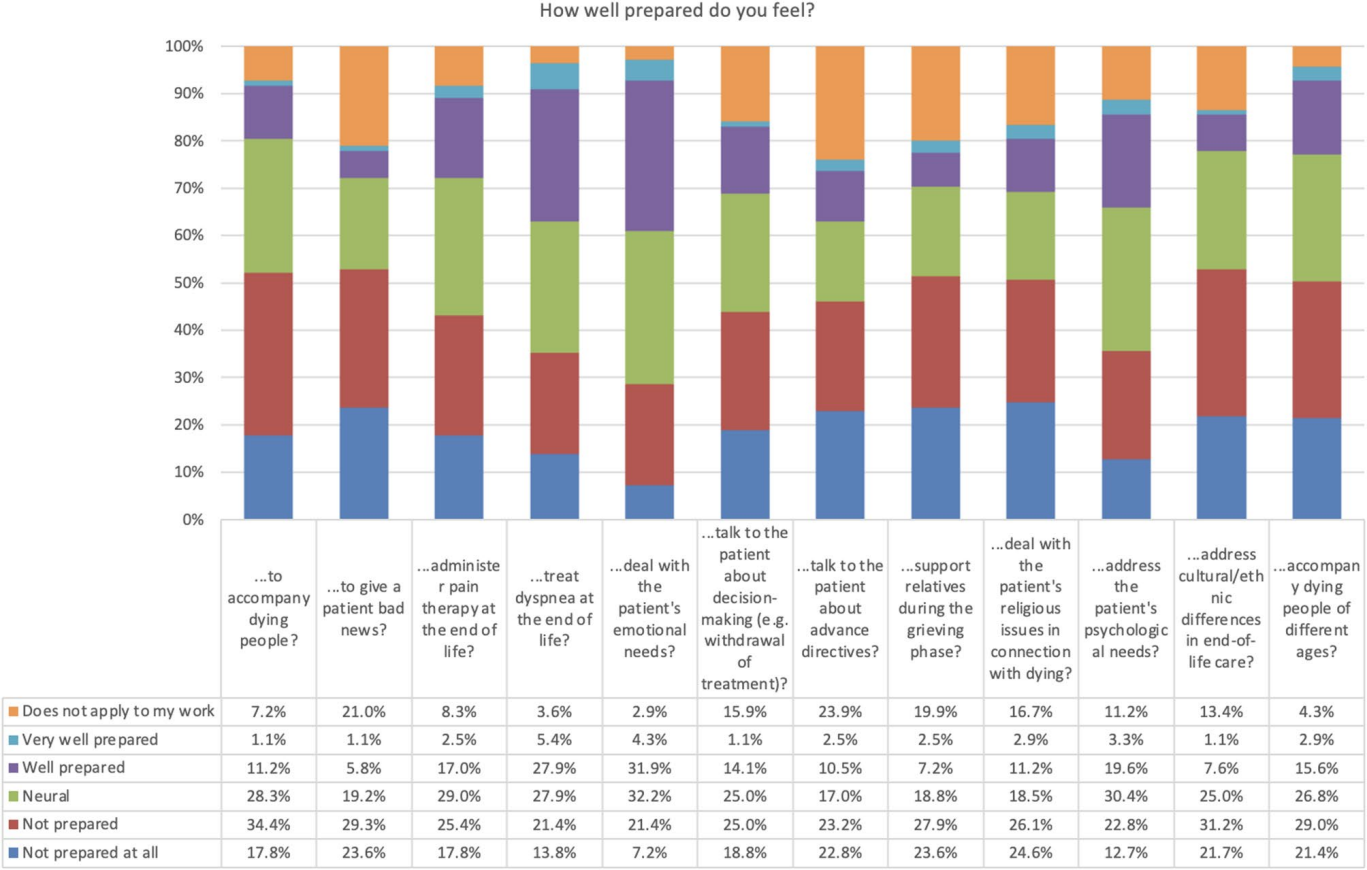
Results of the PCEP-GR Questionnaire

Regarding the emotional impact of working with seriously ill or dying individuals, 17.7% of respondents reported no emotional burden, while 48.9% indicated a sense of burden. Notably, 33.3% of participants expressed neutrality on this matter (see Fig. 1).

**Fig. 2:**
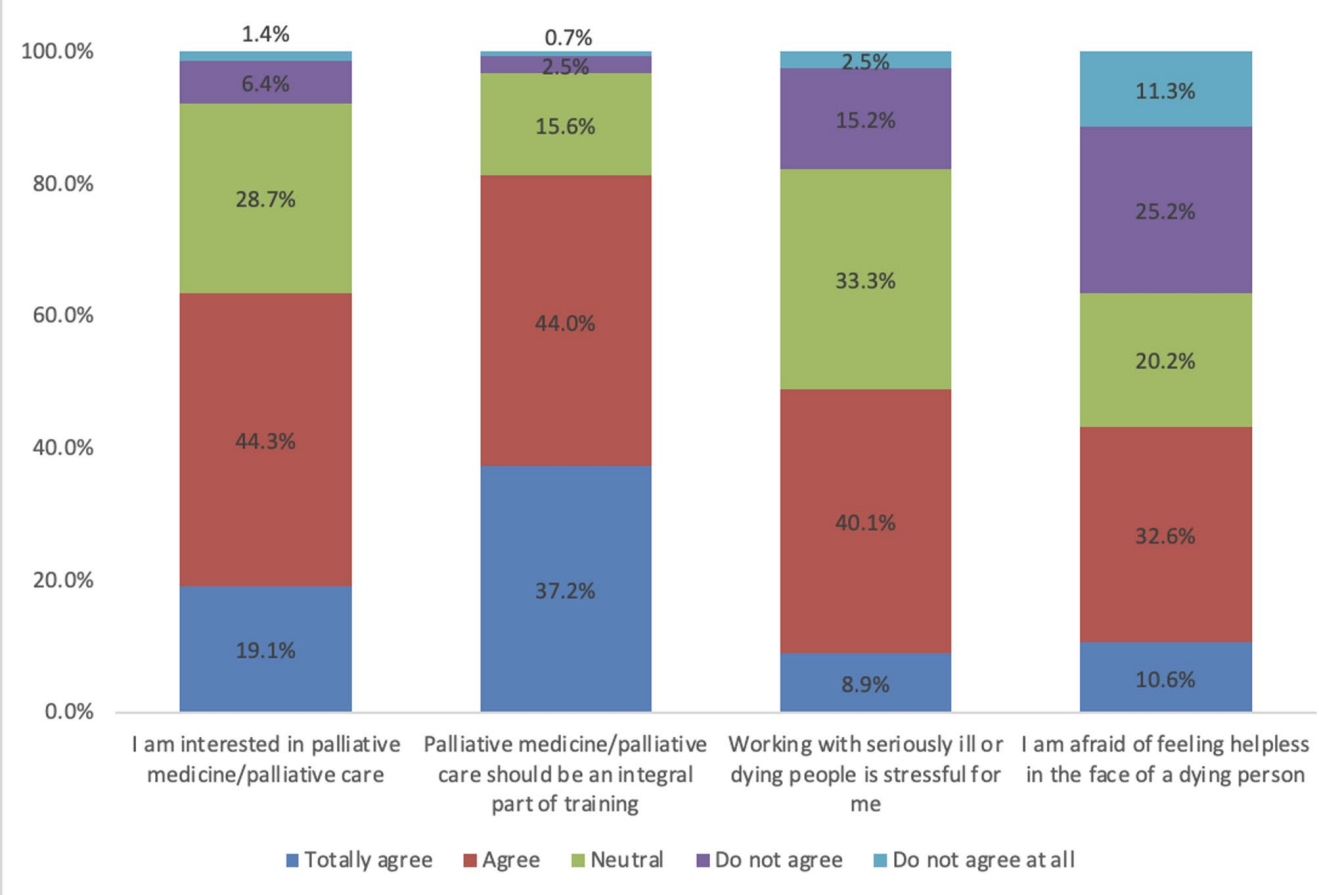
Results of the question “How strongly do you agree with the following statements?”

Furthermore, the data reveals a substantial proportion of respondents, specifically 43.2%, who expressed a sense of agreement with the statement that they feared feeling helpless in the presence of a dying individual. In contrast, 35.5% of respondents expressed disagreement with this statement (see Fig. 2).

### Emotions in End-of-Life care (PCEP-GR)

Among the participants, 243 respondents indicated that they felt “not at all prepared” (22.8%) and “not prepared” (36.8%) when it came to accompanying individuals in the final stages of life. A mere 39 trainees and students (9.6%) indicated that they felt “well prepared”, while only one individual reported feeling “very well prepared”. Regarding the provision of bad news, 250 participants indicated that they felt “not at all prepared” (24.3%) or “not prepared” (36.9%).

Pain management in end-of-life care was identified as a key area of concern: 127 participants (31.1%) reported feeling “not at all prepared”. Similarly, 150 participants (36.8%) reported low levels of preparedness in managing terminal-phase dyspnoea.

When asked about addressing emotional needs, 147 respondents (36.1%) indicated a neutral stance, while 114 (28.0%) reported feeling “well prepared”.

In relation to decision-making processes - particularly those involving treatment withdrawal − 80 participants (19.6%) reported feeling “unprepared”, with 114 (28.0%) adopting a neutral stance.

Support for bereaved relatives was also reported as challenging, with 82 participants (20.1%) indicating they were “not at all prepared”. Similarly, 85 participants (20.8%) reported low preparedness in addressing religious concerns at the end of life.

Preparation for managing psychological needs was rated as neutral by 138 participants (33.9%), while 93 (22.8%) felt “well prepared” in this domain.

Finally, 110 respondents (27.0%) stated they felt “not at all prepared” to accompany dying individuals across different age groups (see Fig. 1 ).

### Existence of and willingness to draw up a living will

Regarding the question of whether they had their own living will, only 28 participants (10.2% of the total sample) answered “yes”. Most of this group had drawn up their living will more than six months ago (75.8%).

The remaining respondents (*n* = 246) stated that they had not yet considered this question (56.1%). Furthermore, 24 respondents (9.7%) indicated that they were not prepared to execute a living will, while 28 (11.3%) expressed the intention to do so within the subsequent 30 days, and 56 (22.7%) within the following six months.

## Discussion

This study offers significant insights into the current status and needs regarding palliative care content in physiotherapy training. The findings unequivocally demonstrate that there are substantial deficiencies in the training, most notably regarding practical experience and theoretical knowledge. This finding aligns with the urgent need for improvement that has been identified in international studies [12–16]. In the following section, the most salient results are contextualized within the extant literature to formulate recommendations for the future design of physiotherapist education.

### Discrepancy between level of knowledge and curricular integration

While a significant proportion of respondents, 76.6%, were able to define palliative care, only a minority, 41.9%, had studied it as part of their training. This discrepancy is consistent with the findings of the EAPC report on the development and availability of palliative care in Europe, which determined that palliative care is inadequately incorporated into European curricula [17]. A similar study by Carrasco et al. showed that less than half of European training programs for healthcare professions contain systematic content on palliative care [6, 18]. In this context, the findings of Woitha et al. demonstrate significant variations in the implementation of palliative care teaching content across Europe. While some countries have already established structured curricula, others demonstrate significant deficiencies. A particularly salient finding is the absence of uniform standards even within individual countries, leading to significant variations in the quality of training [7]. A similar observation was made in the study by Yakasai et al., which revealed significant disparities in the practical implementation of palliative care knowledge in Nigeria, despite a high level of awareness about the subject [19]. This underscores the necessity to incorporate palliative care training into physiotherapy education, as its integration into curricula has been identified as a pivotal factor for enhancing patient care and fostering more effective interprofessional collaboration [7, 19–22]. The integration of curricula addressing ethical aspects, symptom management, and the psychosocial needs of patients has been demonstrated to enhance not only students’ knowledge but also their empathic skills [23]. In the German professional graduation law in physiotherapy, this content is not yet found in comparison to the international physiotherapy graduation.

### Practical experience and emotional stress

Just over half of the participants (51.6%) had practical experience with palliative patients. This figure is notably higher than the results of analogous studies conducted among healthcare professionals, which reported an average practical involvement of 30–40% [24]. However, a significant proportion of respondents, 52.6%, reported experiencing emotional distress while working with terminally ill patients.

This emotional distress is frequently exacerbated by a paucity of preparation for such circumstances [9, 25]. A study by Yongpraderm et al. (2025) demonstrated that internships incorporating supervision and regular reflection sessions can significantly enhance students’ resilience [10]. In a large-scale survey of physiotherapists in Nigeria, Yakasai et al. (2023) found that a high proportion (73.9%) had sufficient knowledge of palliative care, but only 66.7% of them actively implemented palliative care measures in practice. This finding underscores the persistent role of structural and organizational barriers in the implementation of palliative care measures [19].

### Age-dependent spread of living wills and need for education

The low proportion of respondents with a living will (10.2%) aligns with the findings of Wurm et al. (2023), which demonstrate that only a small percentage of individuals complete such documents, particularly among younger population groups. The prevalence of living wills increases significantly with age, while in the early stages of life, there is often uncertainty or a lack of engagement with the topic [26]. Consequently, the incorporation of educational curricula focusing on patient autonomy and rights should be a fundamental component of future training programmes [8, 27].

The results further indicate a need for structured education on end-of-life decision-making. The low completion rate in this young sample, combined with the 22.7% who intended to create a living will within six months, suggests that early curricular exposure can foster awareness and preparedness. Integrating palliative care education that addresses advance directives and patient rights would not only enhance professional competence but also encourage personal reflection among students.

### International perspectives on palliative care in education

On an international level, a range of models illustrate how the integration of palliative care content into healthcare education can be systematically achieved. In the United States of America, the introduction of national standards for palliative care training in medical degree programs has led to a significant improvement in the level of knowledge and professional satisfaction [5]. Furthermore, Wilson et al. (2022) underscore the pivotal role physiotherapists play in nations with advanced palliative care integration, highlighting their multifaceted contributions, including direct patient care, mediation between patients and families, and collaboration with interdisciplinary teams [28].

In German-speaking countries, there is an increasing focus on integrating palliative care into the training of physiotherapists. In Germany, a specific basic curriculum has been developed for therapeutic professional groups, including physiotherapy, which teaches practical physiotherapy procedures in the context of palliative care [29]. Similar concepts can be found in Switzerland, where specialist courses provide in-depth physiotherapy support for patients at the end of life [30] and in Austria, where a university course qualifies specialists in specialized hospice and palliative care [31].

These models demonstrate that systematic integration of education and training not only enhances students’ knowledge and practical skills but also significantly improves the quality of patient care [5, 19, 32–34].

### Treatment volumes and involvement

Data on the actual involvement of physiotherapists in palliative care in Germany are scarce. A recent nationwide survey reported that 77% of physiotherapists had already treated patients with palliative needs, predominantly in hospices, hospital wards and specialised outpatient palliative care teams, where they provided respiratory therapy (85.8%), mobilisation (82.4%) and relaxation techniques (72.9%). Respondents criticised that referrals were often delayed and restricted to short sessions of about 20 min, considered insufficient for complex symptom management [8].

Germany provides around 340 hospital-based palliative care units, 260 adult inpatient hospices, over 1,500 outpatient hospice services, and 367 specialised outpatient palliative care teams, including 36 for children [35]. However, national statistics do not capture the frequency with which physiotherapists are integrated into these services, nor the proportion of patients who receive physiotherapy. In a large outpatient palliative care cohort (*n* = 14,792, 2018–2021), the most common diagnoses were cancer (55%), heart failure (16%), and dementia (8%) [36]. Nevertheless, the rate of physiotherapy involvement in these patients remains unknown.

The paucity of reliable treatment figures has a detrimental effect on workforce planning and training development. It is recommended that future studies quantify the number of palliative patients who receive physiotherapy, the settings in which they receive it, and the treatment intensity. The existence of such evidence would provide a foundation for the allocation of resources, the recognition of professionals, and the systematic integration of palliative care into physiotherapy curricula.

### Recommendations for curriculum development and research

1. Expansion of teaching content: Palliative care should be included as a compulsory part of the curriculum, with a focus on communication, symptom management and ethical decision-making. Specific modules on palliative medicine should be integrated into the academic physiotherapy qualification.
2. Practical training: partnerships between physiotherapy training centres and hospices and palliative care units should be promoted to give students practical experience at an early stage. This should explicitly include opportunities for reflection on farewell rituals, joint case discussions, contact with hospice associations, and structured exchange of experiences, as these elements have been shown to significantly enhance learning outcomes.
3. Supervision and emotional support: supervision and regular reflection should be mandatory components of practical training. Reflection on farewell rituals and the exchange of experiences in group settings should be integrated as specific methods to support students in coping with emotionally demanding situations.
4. Interdisciplinary collaboration: training should promote interprofessional exchange with the aim to strengthen collaboration in palliative care teams. Practical components combined with systematic opportunities for reflection and discussion are therefore crucial for the impact and sustainability of the curriculum.

## Limitations

It is imperative to acknowledge the limitations inherent in the study’s design when interpreting its findings. The sample of 305 participants, which was recruited from various physiotherapy training institutions, could have led to bias and may not be representative of all physiotherapy trainees. The number of trainees in Germany is approximately 21,000 [37, 38]. The data collection process employed a structured online questionnaire, with the understanding that the information provided was self-reported. This method is subject to the potential for respondents to provide socially desirable answers.

Furthermore, the study was constrained to quantitative analyses, which precluded the consideration of qualitative insights into the experiences and needs of trainees and students. This limitation resulted from the study’s focus on numerical data, excluding factors such as teaching methods and the qualifications of teaching staff, which could have provided more comprehensive insights. Furthermore, the timing of data collection may have influenced the perceptions of the participants, as the view of palliative care can change over the course of training.

Another limitation is that no follow-up was conducted to determine which training centres forwarded the invitations, which did not, or the reasons for non-participation. Furthermore, the term ‘palliative care’ was not defined for participants. This may have introduced bias, as different interpretations of the term exist, and providing a uniform definition for all respondents would have ensured greater consistency. In a similar manner, the extent of the reported practical experience was contingent upon the respondents’ comprehension and implementation of the notion of palliative needs. It is important to note that, since this was not explicitly assessed, the results may reflect differences in interpretation rather than actual experience.

The gender distribution of the sample showed most female participants (70.5%). This distribution corresponds to the general gender distribution in physiotherapy, where 73.2% of professionals are female and 26.8% male [31], and thus the generalizability of the results is largely guaranteed. Consequently, the likelihood of a distortion due to gender-specific differences is considered minimal.

## Conclusion

The findings of this study indicate a pressing necessity to methodically incorporate palliative care content into physiotherapy education. The trainees and students exhibited a marked interest in the integration of palliative care, with many already possessing fundamental knowledge yet expressing a desire for more in-depth teaching content and practical experience.

The discrepancy between existing knowledge and practical experience indicates that training in palliative care should be designed to be both theoretical and practical. While the survey provides an overview of current gaps and needs, future research should be supplemented by qualitative approaches to gain deeper insights into the challenges faced by trainees and students and to strengthen the basis for meaningful curricular recommendations. Taken together, these findings provide a robust foundation for the formulation of evidence-based strategies to advance physiotherapy training in this pivotal domain. It is imperative that these developments are systematically evaluated through accompanying research to ensure their efficacy and sustainability.

## Supplementary Information

Supplementary Material 1.

## Data Availability

The data are available upon reasonable request from the authors.
