# Supplementary material for: Palliative care in the education of physiotherapists in Germany - an anonymized cross-sectional survey of trainees and students
Source: BMC Med Educ. 2025 Nov 6;25:1555. doi: 10.1186/s12909-025-08205-4 (PMC12590733; doi:10.1186/s12909-025-08205-4)
Supplement: Supplementary file 1 — Supplementary Material 1. [file 12909_2025_8205_MOESM1_ESM.pdf]

Questionnaire 'Physio_Pall trainees' (raw version, online survey via SurveyMonkey)

Part 1:

1. To what gender do you identify? (m/f/d)
2. What is your age? (________)
3. Training or studies?
4. What year of training or semester are you in? (1/2/3 year; 1-8 semesters).
5. Have you completed any other training in the medical field before training/studying to become a physiotherapist? (Yes/No).
6. Could you explain what palliative medicine/palliative care is? (Yes/No. If "no", then an explanation about palliative care follows so that the rest of the questionnaire can be answered).
7. Do you know what a hospice is? (Yes/No. If "no", then an explanation about hospice care).
8. Have you studied "physiotherapy in palliative medicine/palliative care" in your lessons/studies to date? (Yes/No)
9. If yes,
   - In which year of training or semester? (1/2/3 year; 1-8 semesters)
   - What time frame? (Approx. _____ teaching hours)
   - Did the lessons help you with your training/studies? (Yes/No)
10. If no, would you like to learn something about physiotherapy in palliative medicine/palliative care in your lessons/studies? (Yes/No)
11. Have you worked with palliative care patients during your training/studies? (Yes/No)
12. Did you work with palliative care patients during your training/studies? (Yes/No)
13. If yes,
   - In which area did you work with palliative care patients during your training/studies? (palliative care unit in hospital, other ward in hospital (outside a palliative care unit), hospice, specialised outpatient palliative care service, retirement or nursing home, practice, home visits)
   - How did you find working with palliative care patients (relaxing, rewarding, exhausting, stressful, etc.)
13. If no, would you like to work with palliative care patients? (yes/no)

How much do you agree with the following statements (6-Likert scale):

- I am interested in palliative medicine/palliative care.
- Palliative medicine/palliative care should be an integral part of training.
- Working with seriously ill or dying people is stressful for me.
- I had contact with terminally ill people for the first time during my training.
- I am afraid of feeling helpless in the face of a dying person.

Part 2:

II. PCEP-GR

How well prepared do you feel...	Not prepared at all	Not prepared	Neutral	Well prepared	Very well prepared	Does not apply to my work
1. ...to accompany dying people?	①	②	③	④	⑤	⑥
2. ...to give a patient bad news?	①	②	③	④	⑤	⑥
3. ...provide pain therapy at the end of life?	①	②	③	④	⑤	⑥
4. ...treat dyspnoea at the end of life?	①	②	③	④	⑤	⑥
5. ...deal with the patient's emotional needs?	①	②	③	④	⑤	⑥
6. ...talk to the patient about decision-making (DNR (=do not resuscitate), cancellation of treatment, etc.)?	①	②	③	④	⑤	⑥
7. ...talk to the patient about advance directives?	①	②	③	④	⑤	⑥
8. ...to support relatives in the grieving phase?	①	②	③	④	⑤	⑥
9. ...deal with the patient's religious issues in connection with dying?	①	②	③	④	⑤	⑥
10. ...address the patient's psychological needs?	①	②	③	④	⑤	⑥
11. ...to respond to cultural/ethnic differences in end-of-life care?	①	②	③	④	⑤	⑥
12. ...to accompany dying people of different ages?	①	②	③	④	⑤	⑥

Do you have a living will?			
**Yes** ☐		**No** ☐	
If **yes**, when did you draw up the living will?		If **no**, how ready do you feel to make a living will?	
Within the last 6 months	☐	I haven't thought about it yet	☐
More than 6 months ago	☐	I am not ready to draw up a living will	☐
		I am considering drawing up a living will in the next 6 months	☐
		I am ready to draw up a living will in the next 30 days	☐
